# Exenatide and glucagon co-infusion increases myocardial glucose uptake and improves markers of diastolic dysfunction in adults with type 2 diabetes

**DOI:** 10.1038/s41598-025-04559-3

**Published:** 2025-07-01

**Authors:** James Goodman, Martin Schain, Giovanni Di Stefano, Victoria Lupson, Tracy Horn, Marion Hill, Roie Manavaki, Timothy D. Fryer, Elaine Bumanlag-Amis, Navazh Jalaludeen, Lutz Jermutus, Edvin Johansson, Kerstin Heurling, Henrik Haraldsson, Mark Evans, Joseph Cheriyan, Lars Johansson, Philip Ambery, Ian B. Wilkinson

**Affiliations:** 1 Division of Experimental Medicine and Immunotherapeutics, Department of Medicine, University of Cambridge, Cambridge, UK; 2 Clinical Pharmacology Department, Cambridge University Hospitals NHS Foundation Trust, Cambridge, UK; 3 Antaros Medical AB, Mölndal, Sweden; 4 Department of Clinical Neurosciences, Wolfson Brain Imaging Centre, University of Cambridge, Cambridge, UK; 5 Research and Early Development, Cardiovascular, Renal and Metabolism (CVRM), BioPharmaceuticals R&D, AstraZeneca, Cambridge, UK; 6 Wellcome Trust/MRC Institute of Metabolic Science, University of Cambridge, Cambridge, UK; 7 Cambridge Clinical Trials Unit, Cardiovascular Trials Office, Cambridge University Hospitals NHS Foundation Trust, Cambridge, UK; 8 Late-stage Development, Cardiovascular, Renal and Metabolism (CVRM), BioPharmaceuticals R&D, AstraZeneca, Gothenburg, Sweden

**Keywords:** GLP-1, Glucagon, GLP-1/glucagon, Dual-agonism, Myocardial glucose uptake, ^18^F-FDG, PET MRI, Cardiac MRI, CMR, Cardiovascular diseases, Heart failure, Endocrine system and metabolic diseases, Diabetes, Obesity, Pharmacology, Clinical pharmacology, Pharmacodynamics

## Abstract

Type 2 diabetes (T2D) significantly increases the risk of heart failure, a major cause of hospitalisation and increased morbidity and mortality. Dual and multi-agonist synthetic peptides at the GLP-1 and glucagon receptor are in clinical development as potential new treatments for a range of chronic metabolic conditions including T2D. Here, we aimed to explore the effects of GLP-1 and glucagon dual receptor agonism on myocardial glucose uptake (MGU) and myocardial function in T2D. Eight adults with a mean age of 52 ± 12 years and body mass index 31 ± 4 kg/m^2^ attended three randomised infusion visits using combinations of 0.9% saline, glucagon (12.5 ng/kg/min) and exenatide:glucagon co-infusion (exenatide loading dose 50 ng/min for 30 min then 25 ng/min). MGU and myocardial function were assessed using ^18^F-FDG PET-MRI. MGU increased in *n* = 7/8 (88%) participants from a median of 9.2 × 10^−3^ µmol/g/min (IQR 0.33–19 × 10^−3^ µmol/g/min) with saline, to 20 × 10^−3^ µmol/g/min (5.4–98 × 10^−3^ µmol/g/min) with exenatide:glucagon, *n* = 8, *z* = 2.24, *r =* 0.79, *P* < 0.05. Exenatide:glucagon significantly increased the median left ventricular global peak diastolic circumferential strain rate from 0.619 1/s (0.580–0.716 1/s) to 0.686 1/s (0.644–0.737 1/s) *n* = 8, *z* = 2.37, *r =* 0.84, *P* < 0.05. Left ventricular global longitudinal contraction (as a measure global longitudinal strain) numerically increased by 0.6%, from − 16.0% with saline (-14.0-[-16.7]%) to -16.6% with exenatide:glucagon (-14.1-[-17.6]%), *n* = 8, *z*=-1.54, *r=-*0.54, *P* = 0.123. Further studies are required to explore whether GLP-1/glucagon dual receptor agonists have a role to play in reducing cardiovascular risk and attenuating heart failure related outcomes in patients with chronic metabolic conditions such as T2D.

## Introduction

The heart has the highest oxygen demand in the human body per unit of weight (4.3 mmol/kg/min)^1^. Oxidation of substrates is necessary in order for the heart to generate adenosine triphosphate (ATP). In healthy subjects, under normal physiological conditions, the myocardium depends on the aerobic metabolism of glucose (~ 40%) and β-oxidation of fatty acids (~ 60%), which provides the majority of ATP^2^. The remainder comes from the anaerobic glycolysis of ketone bodies, amino acids and lactate. The choice of substrate metabolism depends on a number of factors including the availability and supply of substrates, oxygen supply and demand, and myocardial workload^3^. Several metabolic changes occur in the diabetic heart including increased fatty acid oxidation, reduced glucose metabolism and loss of metabolic flexibility^4^. Myocardial fatty acid uptake increases by 63% in individuals with impaired glucose tolerance, whereas reduced expression and localisation of glucose transporter 4 (GLUT4), the primary mediator of myocardial glucose uptake (MGU) in T2D, contributes to a 22–41% reduction in MGU compared with non-diabetic individuals^5–9^. The overall shift in substrate metabolism whereby fatty acid oxidation increases and glucose metabolism decreases, despite hyperglycaemia, increases mitochondrial oxygen consumption by around 12% in order to generate the same amount of ATP^10^. These changes in myocardial bioenergetics contribute to the development of diabetic cardiomyopathy, the primary cause of heart failure in type 2 diabetes (T2D)^11^. Diabetic cardiomyopathy is a chronic condition characterised by impairments in cardiac structure and function, in the absence of hypertension, ischemia, or coronary artery disease^12^. Left ventricular (LV) diastolic dysfunction (with a preserved LV ejection fraction) is an early, asymptomatic manifestation of diabetic cardiomyopathy^13^. This progresses to LV dilatation, myocardial fibrosis, LV hypertrophy, and ultimately, symptomatic heart failure with systolic dysfunction and reduced ejection fraction^14^. Epidemiological data from The Framingham Heart Study shows that T2D is an independent risk factor for heart failure, even after adjustment for other cardiac risk factors including age, hypertension, dyslipidaemia, obesity and coronary heart disease^15^.

Dual and multi-agonist synthetic peptides at the GLP-1 and glucagon receptor are currently in clinical development as potential new treatments for T2D and other chronic metabolic conditions including obesity, chronic kidney disease and metabolic dysfunction-associated steatohepatitis^16–19^. Simultaneous targeting of the GLP-1 and glucagon receptors aims to leverage the beneficial metabolic effects of these receptors to suppress appetite and reduce body weight, whilst simultaneously balancing opposing effects on glucose metabolism^20^. With regards to synthetic GLP-1 receptor agonists, a number of landmark trials demonstrate a reduction in major adverse cardiovascular events in T2D and obesity^21–23^. In a meta-analysis of over 60,000 patients, GLP-1 receptor agonists caused a 14% relative risk reduction in major adverse cardiovascular events^24^. GLP-1 receptor agonists improve markers of cardiac function in patients with T2D^25–28^, however their effects on heart failure related outcomes are less certain^29,30^. Data from seven trials indicates that GLP-1 receptor agonists reduce cardiovascular mortality and admissions for heart failure by 9% in T2D^31^. Semaglutide improves heart failure symptoms and reduce heart failure related events in obese individuals with preserved ejection fraction^32^. Other data suggests GLP-1 receptor agonists may have a neutral, or even negative effect, on heart failure related outcomes, especially in those with a reduced ejection fraction (< 40%)^29,33^. By contrast, sodium glucose co-transporter 2 inhibitors (SGLT2i) strongly reduce the risk of hospitalisations for heart failure and cardiovascular death^34,35^. These data have reignited interest in the relationship between anti-diabetic medications and modulating the risk of heart failure in patients with T2D. Despite ongoing clinical development, limited data exists on the effects of dual GLP-1/glucagon receptor agonism on MGU, cardiac function and myocardial energetics. Our aim was to explore this further using ^18^F-FDG (18 F-fluorodeoxyglucose) PET-MRI (positron emission tomography magnetic resonance imaging) in overweight patients with T2D.

## Methods

### Study design

This was a single-centre, single-blinded, physiological, exploratory pilot study. The experimental protocol was approved by South-West-Cornwall and Plymouth Research Ethics Committee (19/SW/0168). The study was registered on Clinicaltrials.gov (NCT04307797) on 13/3/2020 and performed according to the principles of the Declaration of Helsinki.

**Fig. 1:**
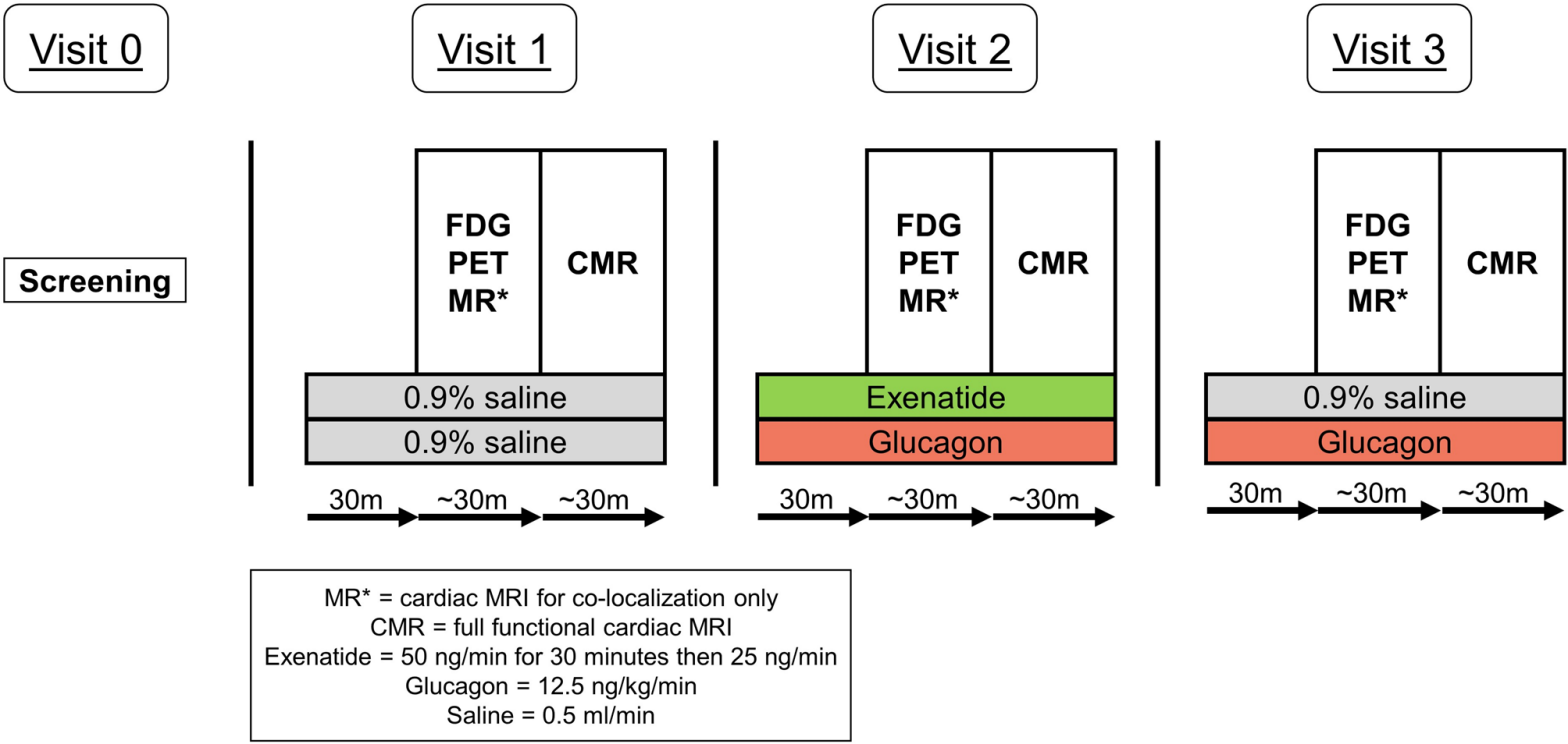
Schematic of study design. Visits 1–3 occurred in a random order as per the randomisation schedule. CMR indicates cardiac MRI; FDG, 18 F-fluorodeoxyglucose; m, minutes; PET, positron emission tomography.

Using a Latin square block design, participants attended three separate imaging visits over a maximum duration of 50 days. Participants received a different intravenous infusion (saline, glucagon or exenatide:glucagon co-infusion) at each visit. Two intravenous cannulae were sited in the upper limbs on the opposite sides – one for the infusion and the other for the ^18^F-FDG injection and blood sampling. Participants received the first 30 min of the intravenous infusion whilst seated before transfer to the PET-MRI scanner. Two pairs of infusions consisting of combinations of saline (Macopharma, France), glucagon (Novo Nordisk, Crawley, 12.5 ng/kg/min) and exenatide (AstraZeneca, UK, loading dose 50 ng/min for 30 min followed by 25 ng/min) were simultaneously infused through a single cannula, via a double lumen extension set, at either 0.5 ml/min or 1.0 ml/min (Braun Perfusor Space pump, Braun, UK) as per the study schematic diagram in Fig. 1. A low-sorb infusion line (Alaris, P7000 extension set, BD, UK) was used for all infusions. Dose selection was based upon previous similar studies^36–45^. In order to simulate the effects of GLP-1 and glucagon receptor dual agonism, exenatide (a licensed GLP-1 receptor agonist which shares 53% amino acid homology with native GLP-1^46^) and glucagon were co-infused.

### Study population

Non-smoking adults with T2D, an elevated BMI (> 25 kg/m^2^) and an HbA1 C < 65 mmol/mol attended an initial screening visit for informed written consent. Females of childbearing potential were excluded. Screening included a medical history, physical examination including anthropometric measurements, vital signs, bloods, 12 lead ECG and a transthoracic echocardiogram. Those with severe hypertension, clinically significant heart disease, chronic kidney disease (creatinine > 150 µmol/L), an active malignancy or type 1 diabetes were excluded from the study. Patients prescribed insulin, GLP-1 receptor agonists or dipeptidyl peptidase 4 inhibitors (DPP4i) were also excluded. Local protocols were followed regarding contraindications for PET and MRI scans including pacemakers and implantable cardioverter-defibrillators.

### Measurements

PET-MRI scans were conducted in the resting supine position following a six hour overnight fast. Participants were asked to avoid strenuous exercise, alcohol, caffeine and energy drinks for 12 h, and anti-inflammatory drugs including aspirin and ibuprofen for 24 h before study visits. Metformin and SGLT2i were withheld on the morning of the study visit. Participants had continuous cardiac monitoring and 30 min ankle BP measurements (Philips Expression MR400, Philips, Netherlands) during the infusion and scan for safety purposes.

## Blood samples

Point of care blood glucose samples (Accu-Check glucose monitor, Roche, France) were taken before starting the infusion to ensure blood glucose < 11.1 mmol/L. If blood glucose > 11.1 mmol/L, the study visit was rescheduled. Blood samples for plasma glucose (for calculation of MGU) and point of care blood glucose were collected prior to the ^18^F-FDG injection (i.e. before the scan) and again, following completion of the PET-MRI scan. Glucose samples were centrifuged at 2000 rpm for two minutes before freezing and overnight storage, due to residual radioactivity in the second glucose sample.

### Imaging methods

Scans were performed on a Signa 3 T PET-MRI scanner (GE Healthcare, Chalfont St Giles, UK) in the Wolfson Brain Imaging Centre (University of Cambridge, Cambridge Biomedical Campus, UK). The first subject was scanned on March 4 th 2022, and the final subject was scanned on October 7 th 2022. For the PET examination, an MR-based attenuation correction scan was obtained before radioligand injection. ^18^F-FDG was administered intravenously, at approximately 2 MBq/kg. Emission data were acquired dynamically over 60 min and reconstructed using VUE Point FXs with 3 iterations and 16 subsets, including all recommended corrections. In cases of excessive patient motion, PET images were reconstructed again with a separate alignment of the attenuation map for each frame following the time at which the motion had occurred. A myocardial region-of-interest was manually delineated on the PET image overlaid on the MRAC image, using both a summation of the early time frames (first five minutes) and the last time frame, enabling a visualization of the myocardial boundaries. The region of interest was typically 5–10 mm thick and placed within the left myocardium. In addition, a region of interest for the blood pool (~ 50–100 voxels) was positioned inside the left ventricle and positioned as far away from the ventricular wall as possible. If excessive motion was detected, regions of interest were dynamically repositioned. The extracted time-activity curves for the myocardium and the blood pool were combined in a Patlak graphical analysis^47^, enabling estimation of myocardial uptake rate of ^18^F-FDG (Ki), which were multiplied by the mean plasma glucose measurements to obtain the MGU.

For the cardiac MRI examination, the protocol consisted of CINE b-SSFP in a stack of short axis (SAX), and in three long axes (LAX) acquired in 2-, 3-, and 4-chamber orientation. These were acquired with 8 mm slice thickness, an acquired in-plane resolution of approximately 1.5 mm, retrospectively reconstructed to 30 cardiac phases. Flow across the mitral valve was quantified using phase contrast MRI. Phase contrast MRI was acquired with 8 mm slice thickness, an acquired in-plane resolution of approximately 2.7 mm, a velocity encoding of 150 cm/s, retrospectively reconstructed to 40 cardiac phases. The SAX and LAX was analysed in Circle CVI42, whereas the mitral flow was quantified in Segment cardiac MRI^48^.

### Statistical analysis

Data were analysed using SPSS version 29 (IBM, New York, USA). No formal power calculations were used given the exploratory nature of the study. Sample size was based on similar experimental pilot studies^37–39^. Imaging data were analysed using Friedman’s Test and the Wilcox Signed-Rank Test. The effect size, r, was calculated by dividing the standardised test statistic, *z*, by the square root of the sample size, *r=*(*z*/√8). Point of care glucose was analysed using area under the curve and the Wilcox Signed-Rank Test. Imaging data and point of care glucose are presented as median ± IQR. A *P* value < 0.05 was deemed significant for all statistical analyses.

### Adverse events

Seven adverse events were reported. Three episodes of urinary frequency in one participant were considered unrelated to the study drugs or PET-MRI scan. Three adverse events of facial tingling secondary to the MRI scan were recorded. One, self-resolving, episode of nausea and tiredness was reported following the combination infusion. All adverse events rapidly resolved. There were no serious adverse events.

## Results

**Fig. 2:**
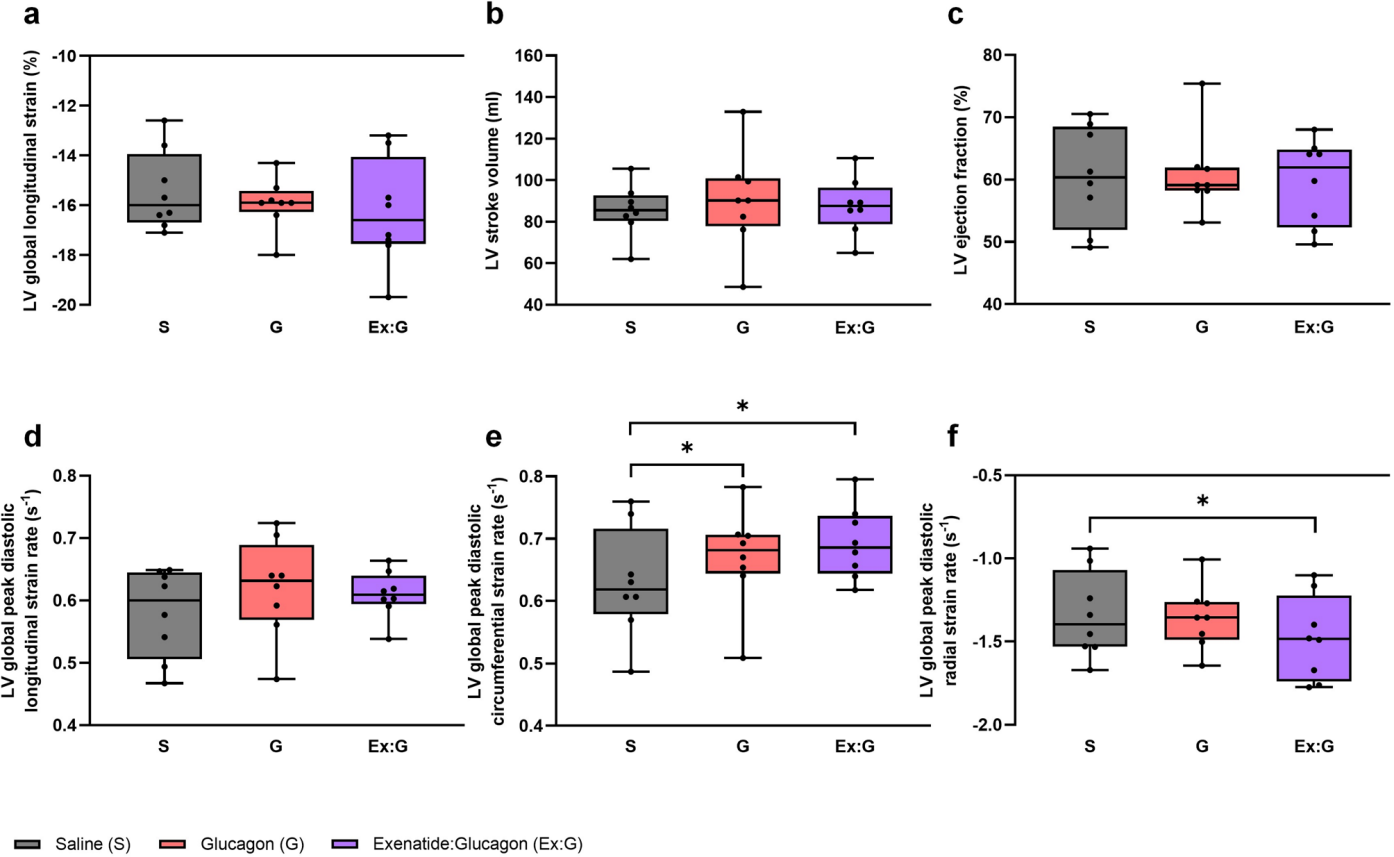
The effect of saline, glucagon and exenatide:glucagon co-infusion on cardiac MRI parameters. a: LV global longitudinal strain. b: LV stroke volume. c: LV ejection fraction. d: LV global peak diastolic longitudinal strain rate. e: LV global peak diastolic circumferential strain rate. f: LV global peak diastolic radial strain rate. Box and whiskers plot with data representing 25 th percentile, median and 75 th percentile. Error bars represent minimum to maximum values. LV indicates left ventricular; S, saline; G, glucagon; Ex: G, exenatide:glucagon combination. *P < 0.05.

**Fig. 3:**
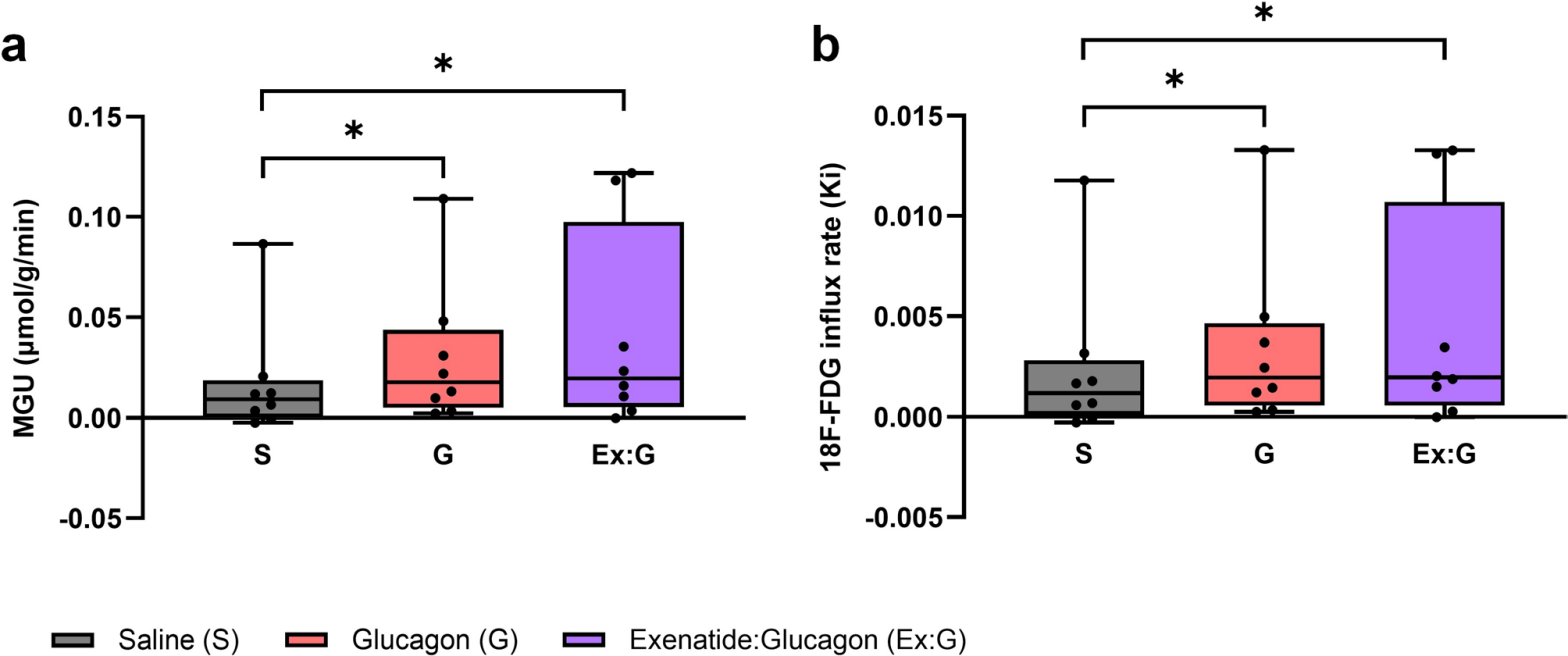
The effect of saline, glucagon and exenatide:glucagon co-infusion on myocardial glucose uptake and the ^18^F-FDG influx rate, Ki. a: myocardial glucose uptake. b: ^18^F-FDG influx rate, Ki. Box and whiskers plot with data representing 25 th percentile, median and 75 th percentile. Error bars represent minimum to maximum values. S represents saline; G, glucagon; Ex: G, exenatide:glucagon combination. *P < 0.05.

**Fig. 4:**
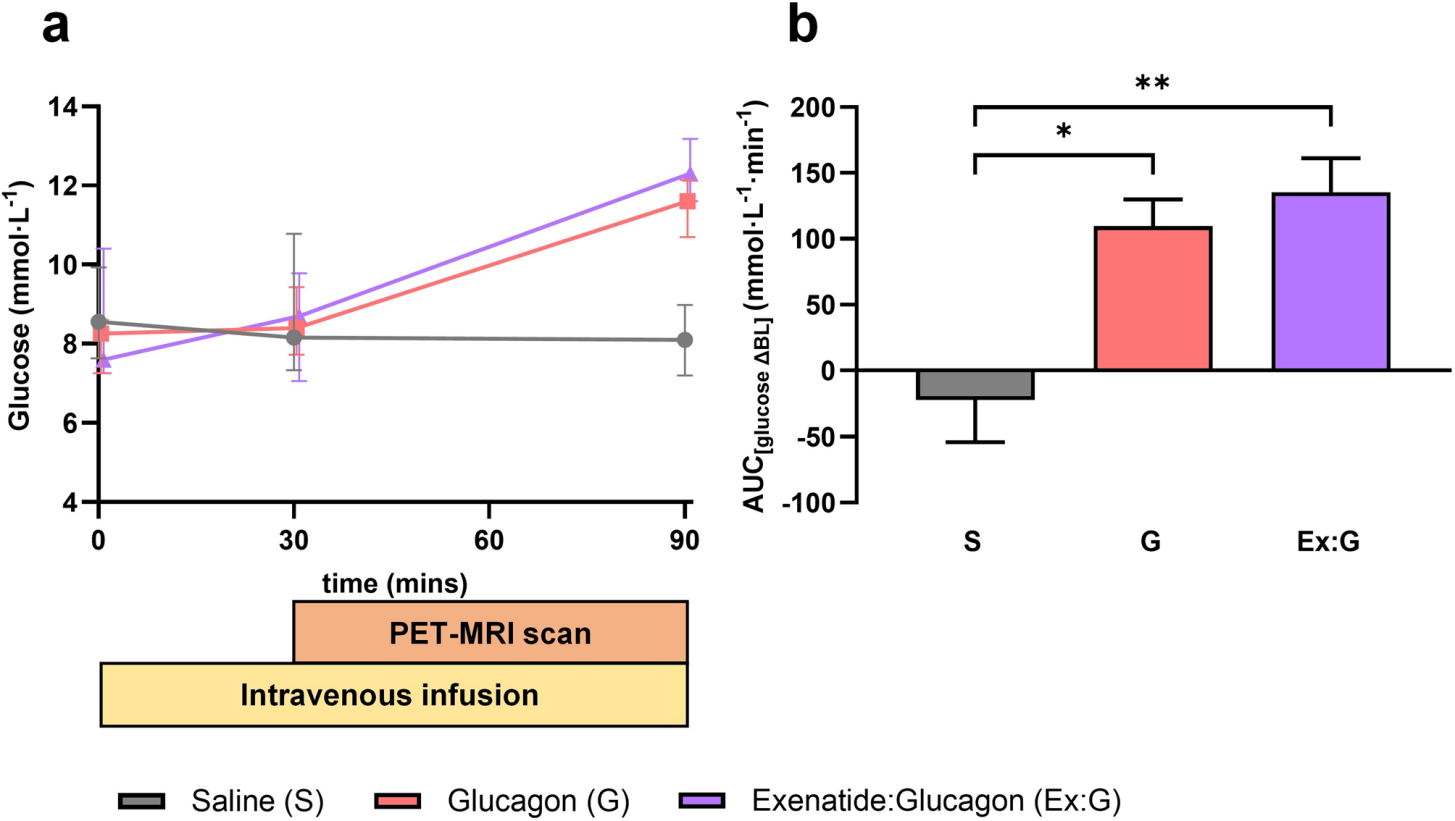
The effect of saline, glucagon and exenatide:glucagon co-infusion on point of care glucose. a: point of care glucose plotted as median ± IQR. b: points of care glucose plotted as change from baseline area under the curve. S represents saline; G, glucagon; Ex: G, exenatide:glucagon combination. *P < 0.05, **P < 0.01.

Eight participants with a mean age of 52 ± 12 years (range 35–73 years) and BMI 31 ± 4 kg/m^2^ completed the study (Table 1). Data are presented in Figs. 2, 3 and 4; Table 2. Unless stated, all statistical comparisons are with saline and data are presented as median (IQR).

**Table 1:** Baseline characteristics, concomitant medications and co-morbidities of the study population. Data indicate unadjusted means ± SD or number (%). ACEi indicates angiotensin converting enzyme; ARB; angiotensin receptor blocker, BMI, body mass index; HR, heart rate; IHD, ischaemic heart disease; SBP, systolic blood pressure; DBP, diastolic blood pressure.

Baseline demographics	
Total (male)	8 (5)
Age (years)	52 ± 12
Age range (years)	35–73
Height (cm)	170 ± 11
Weight (kg)	89 ± 11
BMI (kg/m^2^)	31 ± 4
Body Fat (%)	33 ± 10
Seated SBP (mmHg)	140 ± 13
Seated DBP (mmHg)	79 ± 6
Seated HR (bpm)	70 ± 7
HbA1 C (mmol/mol)	53 ± 7

Comorbidities – N (%)	
Asthma	1 (13)
Autoimmune	1 (13)
Hypertension	4 (50)
Hypercholesterolaemia	6 (75)
IHD	1 (13)
Thyroid disease	2 (25)

Cardiovascular therapies – N (%)	
Antiplatelet	1 (13)
ACEi or ARB	2 (25)
CCB	2 (25)
Statin	7 (88)

Glucose Lowering therapies – N (%)	
Diet control	3 (38)
Metformin	5 (63)
SGLT2i	1 (13)

**Table 2:** Cardiac MRI and PET data for saline, glucagon and exenatide:glucagon co-infusion. Data indicate median (IQR). P-values indicate Friedman’s test. GLS indicates global longitudinal strain; LV, indicates left ventricular; MGU, myocardial glucose uptake.

Cardiac MRI data	Saline	Glucagon	Exenatide:glucagon	*P*-value
LV end systolic volume (ml)	56 (38–79)	59 (44–62)	58 (42–82)	0.882
LV end diastolic volume (ml)	142 (123–161)	142 (126–161)	152 (127–168)	0.882
Left atrial minimum volume (ml)	34 (24–43)	29 (26–35)	31 (25–40)	0.687
Left atrial maximum volume (ml)	74 (55–82)	75 (59–83)	75 (58–84)	0.882
LV stroke volume (ml)	86 (80–93)	90 (78–101)	88 (79–96)	0.607
LV mass (g)	103 (88–118)	115 (99–121)	106 (95–124)	0.093
LV ejection fraction (%)	60.4 (51.9–68.5)	59.1 (58.2–61.9)	62.0 (52.3–64.8)	0.882
LV GLS (%)	−16.0 (−14.0-[−16.7])	−15.9 (−15.4-[−16.3])	−16.6 (−14.1-[−17.6])	0.417
LV global peak systolic GLS rate	−0.781 (−0.649-[−0.955])	−0.842 (−0.684-[−0.967])	−0.766 (−0.720-[−1.003])	0.417
LV global peak diastolic GSL rate	0.600 (0.506–0.645)	0.632 (0.569–0.689)	0.609 (0.594–0.640)	0.446
LV global radial strain (%)	33.6 (26.8–38.5)	31.9 (29.5–38.7)	33.8 (27.6–40.1)	0.687
LV global peak systolic radial strain rate	1.709 (1.470–1.786)	1.740 (1.382–1.934)	1.748 (1.461–1.891)	0.197
LV global peak diastolic radial strain rate	−1.397(−1.070-[−1.531])	−1.356 (−1.261-[−1.490])	−1.484 (−1.223-[−1.740])	*<0.05
LV global circumferential strain (%)	−19.1 (−16.7-[−20.7])	−18.8 (−17.5-[−21.0])	−19.3 (−17.1-[−21.3])	0.291
LV global peak systolic circumferential strain rate	−0.888 (−0.871-[−0.898])	−0.924 (−0.843-[−1.001])	−0.894 (−0.866-[−0.918])	0.882
LV global peak diastolic circumferential strain rate	0.619 (0.580–0.716)	0.682 (0.644–0.707)	0.686 (0.644–0.737)	**<0.01
Ratio between early and late filling (mitral flow)	1.1 (0.87–1.4)	1.1 (0.98–1.3)	1.2 (1.2–1.6)	0.325
Deceleration time (ms)	229 (197–249)	239 (194–258)	198 (186–239)	0.687
Left atrial emptying fraction (%)	52.1 (47.2–60.0)	56.4 (55.3–58.3)	55.4 (45.1–58.9)	0.417

PET data	Saline (x10^−3^)	Glucagon (x10^−3^)	Exenatide:glucagon (x10^−3^)	
MGU (µmol/g/min)	9.2 (0.33–19)	18 (5.1–44)	20 (5.4–98)	0.072
FDG influx rate, Ki	1.1 (0.094–2.8)	2.0 (0.57–4.7)	2.0 (0.58- 11)	0.093

### Glucagon

Glucagon increased MGU in *n* = 7/8 (88%) participants from 9.2 × 10^−3^ µmol/g/min (0.33–19 × 10^−3^ µmol/g/min) with saline, to 18 × 10^−3^ µmol/g/min (5.1–44 × 10^−3^ µmol/g/min) with glucagon, *n* = 8, *z* = 2.10, *r =* 0.74, *P* < 0.05. These differences remained significant following calculation of the ^18^F-FDG influx rate (Ki) (*P* < 0.05). Glucagon significantly increased the LV global peak diastolic circumferential strain rate from 0.619 1/s (0.580–0.716 1/s) to 0.682 1/s (0.644–0.707 1/s) *n* = 8, *z* = 2.10, *r =* 0.74, *P* < 0.05. Stroke volume increased in *n* = 5/8 (63%) participants. There were no significant differences in stroke volume, LV ejection fraction or LV global longitudinal strain between glucagon and saline. Glucagon infusion significantly increased point of care blood glucose (*P* < 0.05).

### Exenatide:glucagon

Exenatide:glucagon increased MGU in *n* = 7/8 (88%) participants from 9.2 × 10^−3^ µmol/g/min (0.33–19 × 10^−3^ µmol/g/min) with saline, to 20 × 10^−3^ µmol/g/min (5.4–98 × 10^−3^ µmol/g/min) with exenatide:glucagon, *n* = 8, *z* = 2.24, *r =* 0.79, *P* < 0.05. These differences remained significant following calculation of the ^18^F-FDG influx rate (Ki) (*P* < 0.05). Exenatide:glucagon co-infusion significantly increased the LV global peak diastolic circumferential strain rate from 0.619 1/s (0.580–0.716 1/s) to 0.686 1/s (0.644–0.737 1/s) *n* = 8, *z* = 2.37, *r =* 0.84, *P* < 0.05. A significant improvement in the LV global peak diastolic radial strain rate from − 1.397 1/s (−1.070-[−1.531] 1/s) to −1.484 1/s (−1.223-[−1.740] 1/s) *n* = 8, *z*=−2.38, *r=-*0.84, *P* < 0.05 was observed. There were no differences in LV ejection fraction between saline, 60.4%, (51.9–68.5%), and exenatide:glucagon, 62.0% (52.3–64.8%), *n* = 8, *z*=−0.84, *r=*−0.30, *P* = 0.401. Exenatide:glucagon increased LV global longitudinal contraction in *n* = 6/8 (75%) participants. Overall, co-infusion increased the longitudinal contraction, as shown by a 0.6% reduction in LV global longitudinal strain from − 16.0% (−14.0-[−16.7]%) to −16.6% (−14.1-[−17.6]%) *n* = 8, *z*=−1.54, *r=*−0.54, *P* = 0.123. Exenatide:glucagon co-infusion significantly increased point of care blood glucose (*P* < 0.05). Exploratory analyses revealed no significant correlation between MGU and HbA1 C, BMI or blood pressure for any of the infusions.

## Discussion

In this human study of overweight adults with T2D, glucagon and exenatide:glucagon co-infusion significantly increased the ^18^F-FDG influx rate (Ki) and MGU. Exenatide:glucagon significantly increased the LV global peak diastolic circumferential strain rate and significantly decreased the LV global peak diastolic radial strain rate. Exenatide:glucagon numerically increased the LV longitudinal contraction as shown by a reduction in LV global longitudinal strain. Glucagon and exenatide: glucagon co-infusion was well tolerated with an acceptable safety profile.

In pre-clinical studies, increases in MGU during an acute coronary event preserves cardiac function^49^. In mice, liraglutide increases myocardial glucose oxidation and alleviates diastolic dysfunction^50^. GLP-1 treatment in rats increases MGU and glucose utilisation by 64% and 14%, respectively^51^. Similar increases in MGU are seen under resting conditions in lean adults (BMI < 25 kg/m^2^) infused with GLP-1 (7–36) amide^52^. No changes in MGU were observed in obese swine and adults with T2D, highlighting the importance of defective insulin signalling in myocardial insulin resistance and T2D^6^. In agreement, the majority of studies show native GLP-1 (7–36) amide and synthetic GLP-1 receptor agonists do not increase MGU in humans across a range of chronic conditions (Table 3). As ^18^F-FDG PET-MRI measures MGU (rather than glucose oxidation), increases in MGU do not necessarily equate to an increase in glucose oxidation^53^. Experimental studies demonstrate that native GLP-1 (7–36) amide improves cardiac function, in pre-clinical and clinical mechanistic studies of ischaemia and reperfusion injury^54–57^. In adults with T2D, lowering average glycaemia is associated with improvements in markers of systolic and diastolic function, regardless of anti-diabetic medication^58^. A meta-analysis of 48 studies shows GLP-1 receptor agonists have a small but beneficial effect on LV ejection fraction (+ 2.6%) in patients with T2D and cardiovascular disease^59^. Physiologically, insulin secretion is enhanced by GLP-1 receptor agonists and is shown to act as a potent stimulator of cardiac glucose metabolism and glucose oxidation^60^. A decrease in insulin resistance secondary to GLP-1 receptor agonists may further optimise myocardial metabolism^61^ and improve cardiac function in patients with T2D^53^. These data indicate that pharmacological interventions which improve myocardial metabolism by switching free fatty acid to glucose oxidation, may drive beneficial improvements in cardiac energetics, contractile function and ultimately, slow the progression of pathological LV remodelling^62^. Changes in MGU and cardiac function are likely to reflect both indirect effects (modulation of inflammation and endothelial function^63^) as well as direct effects on the GLP-1 receptor. Mechanistically, the GLP-1 receptor is widely expressed in the heart and vasculature^64–66^. Hearts from transplant patients and deceased organ donors express GLP-1 mRNA in all four chambers^67^. GLP-1 mRNA receptor transcripts are found in the sino-atrial node^64^ as well as atrial and ventricular cardiomyocytes from normal and ischaemic human hearts^68^. In a large animal experimental model, the GLP-1 receptor is present in pacemaker cells of the sino-atrial node^69^.

**Table 3:** Clinical studies on the effects of native GLP-1 (7–36) amide and synthetic GLP-1 receptor agonists on myocardial glucose uptake. BD indicates twice daily; CO, cardiac output; GLP, glucagon-like peptide-1; HF, heart failure; MP, myocardial perfusion; MBF, myocardial blood flow; MGU, myocardial glucose uptake; MVO_2_, myocardial oxygen consumption; NYHA, new York heat association; OD, once daily; SV, stroke volume; T2D, type 2 diabetes.

Study	Population	Study drugs Duration	PET tracer(s)	Outcome measures	Findings
Gejl et al^103^., 2012	8 males with T2D	Exenatide 1.0 ng/kg/min (0.066 pmol/kg/min) saline	^18^F-FDG ^13^N-ammonia	MGU MBF	Exenatide had no effect on MGU. Exenatide increased MBF by 24% (*p* = 0.009). Linear relationship between the exenatide-induced alterations in MGU and insulin resistance (*p* = 0.010; r^2^ = 0.69).
Moberly et al^52^., 2013	6 lean (saline) 7 lean (GLP-1) 7 T2D and obesity (GLP-1)	GLP-1 (7–36 amide) 1.5 pmol/kg/min saline overnight infusion	^18^F-FDG ^11^C-acetate	MGU MBF MP MVO_2_	GLP-1 increased MGU by x2.8 in lean subjects, compared to untreated lean controls. GLP-1-stimulated rates of MGU in obese adults and T2D were similar to saline control. No differences MP, MVO_2_, blood flow, CO or SV.
Gejl et al^104^., 2014	18 healthy males	GLP-1 (7–36 amide) 1.2 pmol/kg/min i.v. saline 60–360 min	^18^F-FDG	MGU	GLP-1 had no effect on overall MGU. GLP-1 increased MGU in those with a low baseline MGU (the most insulin resistant participants) and reduced MGU in those with a high baseline MGU.
Lepore et al.^105^, 2016	NYHA II, III	Albiglutide 30 mg OD placebo 12 weeks	^18^F-FDG ^11^C-acetate PET	MGU Myocardial efficiency MVO_2_	Albiglutide had no effect on MGU (*p* = 0.59), myocardial efficiency (*p* = 0.90) or MVO_2_ (*p* = 0.25)
Nielsen et al.^106^, 2017	36 HF	Liraglutide 1.8 mg OD placebo 24 weeks	^18^F-FDG ^15^O-H_2_O	MGU MBF Myocardial flow reserve	Liraglutide had no effect MGU (*p* = 0.98), MBF (*p* = 0.76) or myocardial flow reserve (*p* = 0.89)
Chen et al.^107^, 2017	26 T2D with LV dysfunction 10 healthy controls	Exenatide 5 µg BD for 4 weeks then 10 µg BD Insulin glargine 26 weeks	^11^C-acetate [^15^O] H_2_O	MVO_2_ Myocardial efficiency	Exenatide had no effects on cardiac function, perfusion or oxidative metabolism
Mather et al.^108^, 2018	27 T2D	Liraglutide 1.8 mg OD Insulin detemir Insulin detemir: liraglutide 12 weeks	^18^F-FDG ^11^C-acetate ^11^C-palmitate	MGU MBF MP MVO_2_ Fatty acid uptake and oxidation	No differences in MGU, MVO2, fatty acid oxidation, or fatty acid esterification. Liraglutide reduced MBF compared to insulin detemir (*p* = 0.01)

Systemic infusion of glucagon increases heart rate in healthy volunteers, consistent with its well-known chronotropic effect^36,37^. High dose intravenous glucagon, causes a brief inotropic response by increasing cardiac output. This is primarily mediated via an increase in heart rate rather than changes to stroke volume or peripheral vascular resistance^70^. In comparison, lower doses^71^ do not appear to affect cardiac output or mean arterial pressure, suggesting a dose-dependent haemodynamic response^72^. Consistent with this, glucagon did not change LV ejection fraction or stroke volume in this study. Due to its potential inotropic properties, glucagon has long been evaluated as a potential treatment for symptomatic heart failure with data demonstrating mixed clinical outcomes^73^. Drawing conclusions on the clinical efficacy of glucagon in these studies is challenging due to their small size and lack of randomised control arms. The glucagon receptor (and mRNA transcripts) is not present in the sino-atrial node, atria or ventricles of organ donors and explanted hearts, where it also fails to demonstrate any inotropic or chronotropic effects^74^. One proposed mechanism for the above haemodynamic changes is glucagon induced activation of myocardial GLP-1 receptors^70^. Glucagon and native GLP-1 share 47% amino acid homology and have overlapping binding sites. Glucagon has around 140 times less affinity for the GLP-1 receptor (compared with specific GLP-1 agonists) meaning that at high concentrations glucagon may bind and activate the GLP-1 receptor^75–77^. Glucagon is also shown to stimulate catecholamine release from the adrenal gland^78,79^, activate the hypophysis–hypothalamus–adrenal axis^80^ and activate sympathetic activity in the hypothalamus^81^, indicating that some of the cardiovascular effects are conveyed, in part, through activation of the sympathetic nervous system^74^. Physiologically, glucagon promotes glycogenolysis and gluconeogenesis leading to an increase in blood glucose. Compensatory mechanisms increase insulin levels, and both hormones increase fuel availability in the heart^82^. In pre-clinical studies, glucagon and insulin are shown to increase glycolysis and glucose oxidation^83^. We observed a significant increase in MGU with glucagon and this is likely mediated through an increase in glucose availability or a reduction in free fatty acids. By comparison, in individuals without T2D, 0.5 mg intravenous glucagon increased MGU in the myocardium and skeletal muscle, although this was not significant^84^.

Exenatide:glucagon co-infusion significantly increased the ^18^F-FDG influx rate (Ki) and MGU, indicating a potential role of dual agonism in improving myocardial metabolism. With regards to the myocardial function measurements, strain and strain rate represent the magnitude and rate, respectively, of myocardial deformation which provides a quantitive measure of regional and global myocardial function^85^. Global longitudinal strain is a reliable and clinically relevant measure of early subclinical ventricular dysfunction and a predictor of heart failure related events^86^, cardiovascular mortality^87^ and all-cause mortality in patients with heart failure with reduced LV ejection fraction^88^. In patients with heart failure secondary to mitral regurgitation, an increase in LV longitudinal contraction (a 2.3 ± 2% reduction in LV global longitudinal strain) was associated with reduced mortality and hospitalisation rates for heart failure at six months^89^. Six months treatment with semaglutide or dulaglutide significantly improves global longitudinal strain as measured by transthoracic echocardiogram^90^. Exenatide:glucagon co-infusion numerically increased LV longitudinal contraction (0.6% reduction in LV global longitudinal strain) which although not-significant, requires further investigation. Global circumferential strain and global radial strain are two other measurable components of myocardial deformation which are less extensively studied^91^. It should be noted that global radial strain is associated with lower reproducibility compared with other measures^92^. There were no changes in either parameter following glucagon or exenatide:glucagon co-infusion. By contrast, exenatide: glucagon co-infusion significantly increased the LV global peak diastolic circumferential strain rate whereas the LV global peak diastolic radial strain rate significantly decreased. These data indicate that the diastolic changes in both the circumferential and radial direction are quicker which maybe reflective of improved diastolic function^93^. Several small studies report GLP-1 receptor agonists improve LV diastolic dysfunction in patients with T2D^28,94^, however neither of these investigated he effects of GLP-1/glucagon dual agonism. No significant changes in any systolic parameters, LV ejection fraction or stroke volume were observed in this study. We hypothesise that changes in MGU potentially proceed changes cardiac function and as such, measurable improvements of some cardiac parameters are only observed in the medium to long-term. This aligns with previously published data showing that changes in MGU using ^18^F-FDG-PET precede the morphological and mechanical changes in LV systolic dysfunction in db/db mice^95^. However, the relationship between myocardial glucose metabolism and cardiac function during the progression of diabetic cardiomyopathy is less clear^96^. Similarly, cardiac changes maybe harder to detect if the underlying abnormalities are grossly normal, which is broadly the case in this study. These findings warrant further evaluation in larger clinical trials, ideally using novel compounds, to establish whether there is a potential role for dual agonist therapy in treating diabetic cardiomyopathy or heart failure from any cause.

With the exception of glucose, no metabolic data were generated from the study. The authors acknowledge the combination infusion resulted in a greater area under the curve for glucose, compared to the glucagon infusion in isolation, although statistically, there were no differences between the two infusions (Fig. 4). This is likely reflective of the small sample size and the use of point of care glucose sampling, a potential source of pre-analytical and analytical errors^97^ which may also underestimate the true laboratory-based glucose level. From a metabolic perspective, it is evident that glucagon offsets the glucose lowering efficacy of exenatide. This highlights the importance of the respective ratios of GLP-1/glucagon dual agonist compounds for future treatments in metabolic populations, in order to avoid any deleterious effects.

To our knowledge, this is the first human study to explore the effects of GLP: glucagon dual receptor agonism on myocardial function and MGU. Data from haemodynamic early phase clinical trials of GLP-1/glucagon dual agonists demonstrates Cotadutide (MEDI0382)^16,17,98^, Survodutide (BI 456906)^19,99^ and Mazdutide (IBI362)^100^ increase heart rate and reduce blood pressure in patients with T2D or obesity. The GLP-1/glucagon dual agonist Pemvidutide (ALT-801), reduces blood pressure without leading to a clinically significant increase in heart rate^18,101^. Retatrutide, a triple agonist at the GIP, GLP-1 and glucagon receptor increases heart rate and decreases blood pressure in obese adults^102^. In addition to the beneficial effects on glucose metabolism and body weight, GLP-1/glucagon dual receptor agonists are shown to reduce liver glycogen and fat in overweight individuals with T2D^17^ and reduce the urinary albumin-to-creatinine ratio in overweight individuals with T2D and CKD^16^. Further research is required to evaluate these effects to see whether dual agonists improve symptoms and prognosis in patients with diabetic cardiomyopathy and other conditions linked with the metabolic syndrome.

### Limitations

The study is not without limitations. As this was an exploratory pilot study, the sample size was small (*n* = 8). Given the small study numbers, data were not analysed according to gender or so we are unable to exclude this as a confounder. Participants were also on a number of potentially confounding anti-diabetic medications including metformin (*n* = 5) and SGLT2i (*n* = 1). The study involved exposure to ionising radiation and therefore the decision was taken to exclude females of childbearing potential from the study. Observed haemodynamic effects may have been affected by other hormones including insulin and glucose. The glucagon and exenatide:glucagon infusion visits were performed on a different day to the baseline (saline) scan meaning other confounding variables may have influenced the imaging results. The infusion duration was short (150 min), and therefore extrapolating the effects on myocardial function and MGU beyond this time point is not possible. GLP-1 and glucagon receptor occupancy and activation rates differ compared to molecules in clinical development meaning caution should be applied when drawing comparisons with the effects of novel compounds.

The baseline MGU in our study (saline infusion) was 0.009 µmol/g/min. This was significantly lower than other published prior PET studies in T2D in the fasted state (0.13 ± 0.07 µmol/g/min^103^, 0.17 ± 0.05 µmol/g/min^104^, 0.42 ± 0.12 µmol/g/min^8^). The low MGU meant it was challenging to sufficiently delineate the regions of interest with a high degree of certainty. Automated regions of interest analysis were not possible meaning that manually defined regions of interest was applied which can be subjective. This limited the analyses to a single MGU estimate for the entire myocardium, and thus, potential regional changes could not be further explored. In 11 PET scans, patient motion was considerable enough to result in a misalignment between the attenuation map and the emission data meaning re-reconstructed PET images were analysed using the manual region of interest definition. For cardiac MRI, one of the acquired SAX (one visit for one subject) was not analysable due to motion artefacts and therefore imputation using the median was used for statistical analysis.

## Conclusion

This is the first human study to evaluate the effects of dual GLP-1/glucagon agonism on cardiac function and MGU. Exenatide:glucagon co-infusion was well tolerated with a good safety profile. In this pilot study, exenatide:glucagon co-infusion increased MGU and improved the LV global peak diastolic circumferential strain rate and the LV global peak diastolic radial strain rate. Further studies are required to explore whether GLP-1/glucagon dual receptor agonists improve heart failure related outcome measures in patients with T2D.

## Data Availability

The data that support the findings of this study are available from the corresponding author upon reasonable request.

## References

[1] Mudaliar, S., Alloju, S. & Henry, R. R. Can a shift in fuel energetics explain the beneficial cardiorenal outcomes in the EMPA-REG OUTCOME study?? A unifying hypothesis. *Diabetes Care*. **39**, 1115–1122. 10.2337/dc16-0542 (2016). 27289124 10.2337/dc16-0542

[2] Stanley, W. C., Recchia, F. A. & Lopaschuk, G. D. Myocardial substrate metabolism in the normal and failing heart. *Physiol. Rev.* **85**, 1093–1129. 10.1152/physrev.00006.2004 (2005). 15987803 10.1152/physrev.00006.2004

[3] Lopaschuk, G. D., Ussher, J. R., Folmes, C. D. L., Jaswal, J. S. & Stanley, W. C. Myocardial fatty acid metabolism in health and disease. *Physiol. Rev.* **90**, 207–258. 10.1152/physrev.00015.2009 (2010). 20086077 10.1152/physrev.00015.2009

[4] Heather, L. C. & Clarke, K. Metabolism, hypoxia and the diabetic heart. *J. Mol. Cell. Cardiol.* **50**, 598–605. 10.1016/j.yjmcc.2011.01.007 (2011). 21262230 10.1016/j.yjmcc.2011.01.007

[5] Succurro, E. et al. Reduction in global myocardial glucose metabolism in subjects with 1-Hour postload hyperglycemia and impaired glucose tolerance. *Diabetes Care*. **43**, 669–676. 10.2337/dc19-1975 (2020). 31974102 10.2337/dc19-1975

[6] Iozzo, P. et al. Independent association of type 2 diabetes and coronary artery disease with myocardial insulin resistance. *Diabetes* **51**, 3020–3024. 10.2337/diabetes.51.10.3020 (2002). 12351442 10.2337/diabetes.51.10.3020

[7] Kim, G. et al. Visceral adiposity is associated with altered myocardial glucose uptake measured by (18)FDG-PET in 346 subjects with normal glucose tolerance, prediabetes, and type 2 diabetes. *Cardiovasc. Diabetol.* **14**, 148. 10.1186/s12933-015-0310-4 (2015). 26538247 10.1186/s12933-015-0310-4 PMC4632263

[8] Ohtake, T. et al. Myocardial glucose metabolism in noninsulin-dependent diabetes mellitus patients evaluated by FDG-PET. *J. Nucl. Med.* **36**, 456–463 (1995). 7884509

[9] Huynh, K., Bernardo, B. C., McMullen, J. R. & Ritchie, R. H. Diabetic cardiomyopathy: mechanisms and new treatment strategies targeting antioxidant signaling pathways. *Pharmacol. Ther.* **142**, 375–415. 10.1016/j.pharmthera.2014.01.003 (2014). 24462787 10.1016/j.pharmthera.2014.01.003

[10] Bai, J., Liu, C., Zhu, P. & Li, Y. Novel insights into molecular mechanism of mitochondria in diabetic cardiomyopathy. *Front. Physiol.* **11**, 609157. 10.3389/fphys.2020.609157 (2020). 33536936 10.3389/fphys.2020.609157 PMC7849834

[11] Jankauskas, S. S. et al. Heart failure in diabetes. *Metabolism* **125**, 154910. 10.1016/j.metabol.2021.154910 (2021). 34627874 10.1016/j.metabol.2021.154910 PMC8941799

[12] Ritchie, R. H. & Abel, E. D. Basic mechanisms of diabetic heart disease. *Circul. Res.* **126**, 1501–1525. 10.1161/CIRCRESAHA.120.315913 (2020). 10.1161/CIRCRESAHA.120.315913 PMC7251974 32437308

[13] Schannwell, C. M., Schneppenheim, M., Perings, S., Plehn, G. & Strauer, B. E. Left ventricular diastolic dysfunction as an early manifestation of diabetic cardiomyopathy. *Cardiology* **98**, 33–39. 10.1159/000064682 (2002). 12373045 10.1159/000064682

[14] Jia, G., DeMarco, V. G. & Sowers, J. R. Insulin resistance and hyperinsulinaemia in diabetic cardiomyopathy. *Nat. Reviews Endocrinol.* **12**, 144–153. 10.1038/nrendo.2015.216 (2016). 10.1038/nrendo.2015.216 PMC4753054 26678809

[15] Kannel, W. B., Hjortland, M. & Castelli, W. P. Role of diabetes in congestive heart failure: the Framingham study. *Am. J. Cardiol.* **34**, 29–34. 10.1016/0002-9149(74)90089-7 (1974). 4835750 10.1016/0002-9149(74)90089-7

[16] Parker, V. E. R. et al. Efficacy and safety of Cotadutide, a dual glucagon-like peptide-1 and glucagon receptor agonist, in a randomized phase 2a study of patients with type 2 diabetes and chronic kidney disease. *Diabetes Obes. Metab.* **24**, 1360–1369. 10.1111/dom.14712 (2022). 35403793 10.1111/dom.14712 PMC9323481

[17] Parker, V. E. R. et al. Cotadutide promotes glycogenolysis in people with overweight or obesity diagnosed with type 2 diabetes. *Nat. Metab.* **5**, 2086–2093. 10.1038/s42255-023-00938-0 (2023). 38066113 10.1038/s42255-023-00938-0 PMC10730390

[18] KLEIN, S. et al. 334-OR: Pemvidutide (ALT-801), a balanced (1:1) GLP-1/Glucagon dual receptor agonist, induces rapid and marked weight loss without the need for dose Titration in people with overweight/obesity. *Diabetes* **71**10.2337/db22-334-OR (2022).

[19] Blüher, M., Rosenstock, J., Hoefler, J., Manuel, R. & Hennige, A. M. Dose–response effects on HbA1c and bodyweight reduction of survodutide, a dual glucagon/GLP-1 receptor agonist, compared with placebo and open-label semaglutide in people with type 2 diabetes: a randomised clinical trial. *Diabetologia* **67**, 470–482. 10.1007/s00125-023-06053-9 (2024). 38095657 10.1007/s00125-023-06053-9 PMC10844353

[20] Jiang, Y., Zhu, H. & Gong, F. Why does GLP-1 agonist combined with GIP and/or GCG agonist have greater weight loss effect than GLP-1 agonist alone in obese adults without type 2 diabetes? Diabetes. *Obes. Metabolism*. **27**, 1079–1095. 10.1111/dom.16106 (2025). 10.1111/dom.16106 39592891

[21] Marx, N., Husain, M., Lehrke, M., Verma, S. & Sattar, N. GLP-1 receptor agonists for the reduction of atherosclerotic cardiovascular risk in patients with type 2 diabetes. *Circulation* **146**, 1882–1894. 10.1161/CIRCULATIONAHA.122.059595 (2022). 36508493 10.1161/CIRCULATIONAHA.122.059595

[22] Singh, S. et al. Safety and efficacy of glucagon-like peptide-1 receptor agonists on cardiovascular events in overweight or obese non-diabetic patients. *Curr. Probl. Cardiol.* **49**, 102403. 10.1016/j.cpcardiol.2024.102403 (2024). 38237815 10.1016/j.cpcardiol.2024.102403

[23] Lincoff, A. M. et al. Semaglutide and cardiovascular outcomes in obesity without diabetes. *N Engl. J. Med.* **389**, 2221–2232. 10.1056/NEJMoa2307563 (2023). 37952131 10.1056/NEJMoa2307563

[24] Sattar, N. et al. Cardiovascular, mortality, and kidney outcomes with GLP-1 receptor agonists in patients with type 2 diabetes: a systematic review and meta-analysis of randomised trials. *Lancet Diabetes Endocrinol.* **9**, 653–662. 10.1016/s2213-8587(21)00203-5 (2021). 34425083 10.1016/S2213-8587(21)00203-5

[25] Lambadiari, V. et al. Effects of 6-month treatment with the glucagon like peptide-1 analogue liraglutide on arterial stiffness, left ventricular myocardial deformation and oxidative stress in subjects with newly diagnosed type 2 diabetes. *Cardiovasc. Diabetol.* **17**, 8. 10.1186/s12933-017-0646-z (2018). 29310645 10.1186/s12933-017-0646-z PMC5759220

[26] Scalzo, R. L. et al. Exenatide improves diastolic function and attenuates arterial stiffness but does not alter exercise capacity in individuals with type 2 diabetes. *J. Diabetes Complications*. **31**, 449–455. 10.1016/j.jdiacomp.2016.10.003 (2017). 27884660 10.1016/j.jdiacomp.2016.10.003 PMC5787373

[27] Saponaro, F. et al. Improved diastolic function in type 2 diabetes after a six month liraglutide treatment. *Diabetes Res. Clin. Pract.* **118**, 21–28. 10.1016/j.diabres.2016.04.046 (2016). 27485853 10.1016/j.diabres.2016.04.046

[28] Bizino, M. B. et al. Effect of liraglutide on cardiac function in patients with type 2 diabetes mellitus: randomized placebo-controlled trial. *Cardiovasc. Diabetol.* **18**, 55. 10.1186/s12933-019-0857-6 (2019). 31039778 10.1186/s12933-019-0857-6 PMC6492440

[29] Khan, M. S. et al. Glucagon-Like peptide 1 receptor agonists and heart failure. *Circulation* **142**, 1205–1218. 10.1161/CIRCULATIONAHA.120.045888 (2020). 32955939 10.1161/CIRCULATIONAHA.120.045888

[30] Natali, A., Nesti, L., Tricò, D. & Ferrannini, E. Effects of GLP-1 receptor agonists and SGLT-2 inhibitors on cardiac structure and function: a narrative review of clinical evidence. *Cardiovasc. Diabetol.* **20**, 196. 10.1186/s12933-021-01385-5 (2021). 34583699 10.1186/s12933-021-01385-5 PMC8479881

[31] Kristensen, S. L. et al. Cardiovascular, mortality, and kidney outcomes with GLP-1 receptor agonists in patients with type 2 diabetes: a systematic review and meta-analysis of cardiovascular outcome trials. *Lancet Diabetes Endocrinol.* **7**, 776–785. 10.1016/s2213-8587(19)30249-9 (2019). 31422062 10.1016/S2213-8587(19)30249-9

[32] Kosiborod, M. N. et al. Semaglutide in patients with heart failure with preserved ejection fraction and obesity. *N Engl. J. Med.* **389**, 1069–1084. 10.1056/NEJMoa2306963 (2023). 37622681 10.1056/NEJMoa2306963

[33] Neves, J. S., Packer, M. & Ferreira, J. P. Increased risk of heart failure hospitalization with GLP-1 receptor agonists in patients with reduced ejection fraction: A Meta-Analysis of the EXSCEL and FIGHT trials. *J. Card Fail.* **29**, 1107–1109. 10.1016/j.cardfail.2023.03.017 (2023). 37028749 10.1016/j.cardfail.2023.03.017

[34] Vaduganathan, M. et al. SGLT-2 inhibitors in patients with heart failure: a comprehensive meta-analysis of five randomised controlled trials. *Lancet* **400**, 757–767. 10.1016/s0140-6736(22)01429-5 (2022). 36041474 10.1016/S0140-6736(22)01429-5

[35] Solomon, S. D. et al. Dapagliflozin in heart failure with mildly reduced or preserved ejection fraction. *N Engl. J. Med.* **387**, 1089–1098. 10.1056/NEJMoa2206286 (2022). 36027570 10.1056/NEJMoa2206286

[36] Salem, V. et al. Glucagon increases energy expenditure independently of brown adipose tissue activation in humans. *Diabetes Obes. Metab.* **18**, 72–81. 10.1111/dom.12585 (2016). 26434748 10.1111/dom.12585 PMC4710848

[37] Tan, T. M. et al. Coadministration of glucagon-like peptide-1 during glucagon infusion in humans results in increased energy expenditure and amelioration of hyperglycemia. *Diabetes* **62**, 1131–1138. 10.2337/db12-0797 (2013). 23248172 10.2337/db12-0797 PMC3609580

[38] Cegla, J. et al. Coinfusion of low-dose GLP-1 and glucagon in man results in a reduction in food intake. *Diabetes* **63**, 3711–3720. 10.2337/db14-0242 (2014). 24939425 10.2337/db14-0242

[39] Bagger, J. I. et al. Effect of Oxyntomodulin, glucagon, GLP-1, and combined Glucagon + GLP-1 infusion on food intake, appetite, and resting energy expenditure. *J. Clin. Endocrinol. Metab.* **100**, 4541–4552. 10.1210/jc.2015-2335 (2015). 26445112 10.1210/jc.2015-2335

[40] Smits, M. M. et al. GLP-1 receptor agonist exenatide increases capillary perfusion independent of nitric oxide in healthy overweight men. Arteriosclerosis, thrombosis. *Vascular Biology*. **35**, 1538–1543. 10.1161/atvbaha.115.305447 (2015). 10.1161/ATVBAHA.115.305447 25908765

[41] Smits, M. M. et al. Exenatide acutely increases heart rate in parallel with augmented sympathetic nervous system activation in healthy overweight males. *Br. J. Clin. Pharmacol.* **81**, 613–620. 10.1111/bcp.12843 (2016). 26609792 10.1111/bcp.12843 PMC4799913

[42] Smits, M. M. et al. GLP-1-Based therapies have no microvascular effects in type 2 diabetes mellitus: an acute and 12-Week randomized, Double-Blind, Placebo-Controlled trial. *Arterioscler. Thromb. Vasc. Biol.* **36**, 2125–2132. 10.1161/atvbaha.116.307930 (2016). 27562916 10.1161/ATVBAHA.116.307930

[43] Smits, M. M. et al. Heart rate acceleration with GLP-1 receptor agonists in type 2 diabetes patients: an acute and 12-week randomised, double-blind, placebo-controlled trial. *Eur. J. Endocrinol.* **176**, 77–86. 10.1530/eje-16-0507 (2017). 27777261 10.1530/EJE-16-0507

[44] Nathanson, D. et al. Effects of intravenous exenatide in type 2 diabetic patients with congestive heart failure: a double-blind, randomised controlled clinical trial of efficacy and safety. *Diabetologia* **55**, 926–935. 10.1007/s00125-011-2440-x (2012). 22246377 10.1007/s00125-011-2440-x

[45] Nathanson, D., Frick, M., Ullman, B. & Nyström, T. Exenatide infusion decreases atrial natriuretic peptide levels by reducing cardiac filling pressures in type 2 diabetes patients with decompensated congestive heart failure. *Diabetol. Metab. Syndr.* **8**, 5. 10.1186/s13098-015-0116-2 (2016). 26759609 10.1186/s13098-015-0116-2 PMC4709886

[46] Koliaki, C. & Doupis, J. Incretin-based therapy: a powerful and promising weapon in the treatment of type 2 diabetes mellitus. *Diabetes Therapy*. **2**, 101–121. 10.1007/s13300-011-0002-3 (2011). 22127804 10.1007/s13300-011-0002-3 PMC3144767

[47] Patlak, C. S., Blasberg, R. G. & Fenstermacher, J. D. Graphical evaluation of Blood-to-Brain transfer constants from Multiple-Time uptake data. *J. Cereb. Blood Flow. Metabolism*. **3**, 1–7. 10.1038/jcbfm.1983.1 (1983). 10.1038/jcbfm.1983.1 6822610

[48] Heiberg, E. et al. Design and validation of Segment - freely available software for cardiovascular image analysis. *BMC Med. Imaging*. **10**, 1. 10.1186/1471-2342-10-1 (2010). 20064248 10.1186/1471-2342-10-1 PMC2822815

[49] Owen, P., Dennis, S. & Opie, L. H. Glucose flux rate regulates onset of ischemic contracture in globally underperfused rat hearts. *Circ. Res.* **66**, 344–354 (1990). 2297807 10.1161/01.res.66.2.344

[50] Almutairi, M. et al. The GLP-1 receptor agonist liraglutide increases myocardial glucose oxidation rates via indirect mechanisms and mitigates experimental diabetic cardiomyopathy. *Can. J. Cardiol.* **37**, 140–150. 10.1016/j.cjca.2020.02.098 (2021). 32640211 10.1016/j.cjca.2020.02.098

[51] Aravindhan, K. et al. Cardioprotection resulting from Glucagon-Like Peptide-1 administration involves shifting metabolic substrate utilization to increase energy efficiency in the rat heart. *PloS One*. **10**, e0130894–e0130894. 10.1371/journal.pone.0130894 (2015). 26098939 10.1371/journal.pone.0130894 PMC4476748

[52] Moberly, S. P. et al. Impaired cardiometabolic responses to glucagon-like peptide 1 in obesity and type 2 diabetes mellitus. *Basic Res. Cardiol.* **108**, 365. 10.1007/s00395-013-0365-x (2013). 23764734 10.1007/s00395-013-0365-x PMC3731771

[53] Al Batran, R., Almutairi, M. & Ussher, J. R. Glucagon-like peptide-1 receptor mediated control of cardiac energy metabolism. *Peptides* **100**, 94–100. 10.1016/j.peptides.2017.12.005 (2018). 29412838 10.1016/j.peptides.2017.12.005

[54] Sokos, G. G., Nikolaidis, L. A., Mankad, S., Elahi, D. & Shannon, R. P. Glucagon-like peptide-1 infusion improves left ventricular ejection fraction and functional status in patients with chronic heart failure. *J. Card Fail.* **12**, 694–699. 10.1016/j.cardfail.2006.08.211 (2006). 17174230 10.1016/j.cardfail.2006.08.211

[55] Read, P. A., Khan, F. Z. & Dutka, D. P. Cardioprotection against ischaemia induced by Dobutamine stress using glucagon-like peptide-1 in patients with coronary artery disease. *Heart (British Cardiac Society)*. **98**, 408–413. 10.1136/hrt.2010.219345 (2012). 21561896 10.1136/hrt.2010.219345

[56] Sokos, G. G. et al. Effect of glucagon-like peptide-1 (GLP-1) on glycemic control and left ventricular function in patients undergoing coronary artery bypass grafting. *Am. J. Cardiol.* **100**, 824–829. 10.1016/j.amjcard.2007.05.022 (2007). 17719327 10.1016/j.amjcard.2007.05.022

[57] Nikolaidis, L. A. et al. Recombinant glucagon-like peptide-1 increases myocardial glucose uptake and improves left ventricular performance in conscious dogs with pacing-induced dilated cardiomyopathy. *Circulation* **110**, 955–961. 10.1161/01.Cir.0000139339.85840.Dd (2004). 15313949 10.1161/01.CIR.0000139339.85840.DD

[58] Leung, M., Wong, V. W., Hudson, M. & Leung, D. Y. Impact of improved glycemic control on cardiac function in type 2 diabetes mellitus. *Circ. Cardiovasc. Imaging*. **9**, e003643. 10.1161/CIRCIMAGING.115.003643 (2016). 26962125 10.1161/CIRCIMAGING.115.003643

[59] Zhang, D-P., Xu, L., Wang, L-F., Wang, H-J. & Jiang, F. Effects of antidiabetic drugs on left ventricular function/dysfunction: a systematic review and network meta-analysis. *Cardiovasc. Diabetol.* **19**, 10. 10.1186/s12933-020-0987-x (2020). 31969144 10.1186/s12933-020-0987-x PMC6977298

[60] Ussher, J. R. et al. Insulin-stimulated cardiac glucose oxidation is increased in high-fat diet-induced obese mice lacking malonyl coa decarboxylase. *Diabetes* **58**, 1766–1775. 10.2337/db09-0011 (2009). 19478144 10.2337/db09-0011 PMC2712785

[61] Ussher, J. R. & Drucker, D. J. Cardiovascular actions of incretin-based therapies. *Circ. Res.* **114**, 1788–1803. 10.1161/circresaha.114.301958 (2014). 24855202 10.1161/CIRCRESAHA.114.301958

[62] Ashrafian, H., Frenneaux, M. P. & Opie, L. H. Metabolic mechanisms in heart failure. *Circulation* **116**, 434–448. 10.1161/circulationaha.107.702795 (2007). 17646594 10.1161/CIRCULATIONAHA.107.702795

[63] Górriz, J. L. et al. GLP-1 receptor agonists and diabetic kidney disease: A call of attention to nephrologists. *J. Clin. Med.* **9**10.3390/jcm9040947 (2020). 10.3390/jcm9040947 PMC7231090 32235471

[64] Kanemaru, K. et al. Spatially resolved multiomics of human cardiac niches. *Nature* **619**, 801–810. 10.1038/s41586-023-06311-1 (2023). 37438528 10.1038/s41586-023-06311-1 PMC10371870

[65] Helmstädter, J. et al. Glucagon-like peptide-1 (GLP-1) receptor agonists and their cardiovascular benefits—The role of the GLP-1 receptor. *Br. J. Pharmacol.* **179**, 659–676. 10.1111/bph.15462 (2022). 33764504 10.1111/bph.15462 PMC8820186

[66] Almutairi, M., Al Batran, R. & Ussher, J. R. Glucagon-like peptide-1 receptor action in the vasculature. *Peptides* **111**, 26–32. 10.1016/j.peptides.2018.09.002 (2019). 30227157 10.1016/j.peptides.2018.09.002

[67] Baggio, L. L. et al. GLP-1 receptor expression within the human heart. *Endocrinology* **159**, 1570–1584. 10.1210/en.2018-00004 (2018). 29444223 10.1210/en.2018-00004 PMC5939638

[68] McLean, B. A., Wong, C. K., Kabir, M. G. & Drucker, D. J. Glucagon-like Peptide-1 receptor Tie2 + cells are essential for the cardioprotective actions of liraglutide in mice with experimental myocardial infarction. *Mol. Metab.* **66**, 101641. 10.1016/j.molmet.2022.101641 (2022). 36396031 10.1016/j.molmet.2022.101641 PMC9706177

[69] Lubberding, A. F. et al. Glucagon-like peptide-1 increases heart rate by a direct action on the sinus node. *Cardiovasc. Res.* **120**, 1427–1441. 10.1093/cvr/cvae120 (2024). 38832935 10.1093/cvr/cvae120 PMC11472427

[70] Petersen, K. M. et al. High-Dose glucagon has hemodynamic effects regardless of cardiac Beta-Adrenoceptor Blockade: A randomized clinical trial. *J. Am. Heart Assoc.* **9**, e016828. 10.1161/jaha.120.016828 (2020). 33103603 10.1161/JAHA.120.016828 PMC7763418

[71] Klein, S. W., Morch, J. E. & Mahon, W. A. Cardiovascular effects of glucagon in man. *Can. Med. Assoc. J.* **98**, 1161–1164 (1968). 5657181 PMC1924287

[72] Thuesen, L., Christiansen, J. S., Sorensen, K. E., Orskov, H. & Henningsen, P. Low-dose intravenous glucagon has no effect on myocardial contractility in normal man. An echocardiographic study. *Scand. J. Clin. Lab. Investig.* **48**, 71–75 (1988). 3064277 10.3109/00365518809085396

[73] Nord, H. J., Fontanes, A. L. & Williams, J. F. Jr Treatment of congestive heart failure with glucagon. *Ann. Intern. Med.* **72**, 649–653 (1970). 4915149 10.7326/0003-4819-72-5-649

[74] Aranda-Domene, R., Orenes-Piñero, E., Arribas-Leal, J. M., Canovas-Lopez, S. & Hernández-Cascales, J. Evidence for a lack of inotropic and chronotropic effects of glucagon and glucagon receptors in the human heart. *Cardiovasc. Diabetol.* **22**, 128. 10.1186/s12933-023-01859-8 (2023). 37254135 10.1186/s12933-023-01859-8 PMC10230788

[75] Runge, S., Wulff, B. S., Madsen, K., Bräuner-Osborne, H. & Knudsen, L. B. Different domains of the glucagon and glucagon-like peptide-1 receptors provide the critical determinants of ligand selectivity. *Br. J. Pharmacol.* **138**, 787–794. 10.1038/sj.bjp.0705120 (2003). 12642379 10.1038/sj.bjp.0705120 PMC1573731

[76] Knudsen, L. B. et al. Small-molecule agonists for the glucagon-like peptide 1 receptor. Proceedings of the National Academy of Sciences 104:937–942 doi: (2007). 10.1073/pnas.0605701104 10.1073/pnas.0605701104 PMC1783418 17213325

[77] Neumann, J., Hofmann, B., Dhein, S. & Gergs, U. Glucagon and its receptors in the mammalian heart. *Int. J. Mol. Sci.* **24**10.3390/ijms241612829 (2023). 10.3390/ijms241612829 PMC10454195 37629010

[78] Koshika, T., Nagayama, T., Kimura, T. & Satoh, S. Glucagon facilitates adrenal catecholamine release mediated by nicotinic receptors but not by muscarinic receptors in anesthetized dogs. *J. Cardiovasc. Pharmacol.* **28**, 585–590. 10.1097/00005344-199610000-00017 (1996). 8891886 10.1097/00005344-199610000-00017

[79] Chernow, B. et al. Glucagon: endocrine effects and calcium involvement in cardiovascular actions in dogs. *Circ. Shock*. **19**, 393–407 (1986). 2427245

[80] Arvat, E. et al. Glucagon is an ACTH secretagogue as effective as hCRH after intramuscolar administration while it is ineffective when given intravenously in normal subjects. *Pituitary* **3**, 169–173. 10.1023/a:1011451710004 (2000). 11383481 10.1023/a:1011451710004

[81] Shimizu, H., Egawa, M., Yoshimatsu, H. & Bray, G. A. Glucagon injected in the lateral hypothalamus stimulates sympathetic activity and suppresses monoamine metabolism. *Brain Res.* **630**, 95–100. 10.1016/0006-8993(93)90647-6 (1993). 8118708 10.1016/0006-8993(93)90647-6

[82] Ceriello, A., Genovese, S., Mannucci, E. & Gronda, E. Glucagon and heart in type 2 diabetes: new perspectives. *Cardiovasc. Diabetol.* **15**, 123. 10.1186/s12933-016-0440-3 (2016). 27568179 10.1186/s12933-016-0440-3 PMC5002329

[83] Ali, S. & Drucker, D. J. Benefits and limitations of reducing glucagon action for the treatment of type 2 diabetes. *Am. J. Physiol. Endocrinol. Metab.* **296**, E415–421. 10.1152/ajpendo.90887.2008 (2009). 19116373 10.1152/ajpendo.90887.2008

[84] Yasuda, S. et al. The effect of glucagon on FDG uptake in skeletal muscle. *Tokai J. Exp. Clin. Med.* **37**, 11–13 (2012). 22488557

[85] Hoit, B. D. Strain and strain rate echocardiography and coronary artery disease. *Circ. Cardiovasc. Imaging*. **4**, 179–190. 10.1161/CIRCIMAGING.110.959817 (2011). 21406664 10.1161/CIRCIMAGING.110.959817

[86] Janwanishstaporn, S. et al. Prognostic value of global longitudinal strain in patients with heart failure with improved ejection fraction. *JACC: Heart Fail.* **10**, 27–37. 10.1016/j.jchf.2021.08.007 (2022). 34969494 10.1016/j.jchf.2021.08.007

[87] Biering-Sørensen, T. et al. Global longitudinal strain by echocardiography predicts Long-Term risk of cardiovascular morbidity and mortality in a Low-Risk general population: the Copenhagen City heart study. *Circ. Cardiovasc. Imaging*. **10**10.1161/circimaging.116.005521 (2017). 10.1161/CIRCIMAGING.116.005521 PMC5363277 28264868

[88] Sengeløv, M. et al. Global longitudinal strain is a superior predictor of All-Cause mortality in heart failure with reduced ejection fraction. *JACC Cardiovasc. Imaging*. **8**, 1351–1359. 10.1016/j.jcmg.2015.07.013 (2015). 26577264 10.1016/j.jcmg.2015.07.013

[89] Pio, S. M. et al. Changes in left ventricular global longitudinal strain in patients with heart failure and secondary mitral regurgitation: the COAPT trial. *J. Am. Heart Association*. **12**, e029956. 10.1161/JAHA.122.029956 (2023). 10.1161/JAHA.122.029956 PMC10547326 37646214

[90] Basile, P. et al. Improvement of left ventricular global longitudinal strain after 6-Month therapy with GLP-1RAs semaglutide and dulaglutide in type 2 diabetes mellitus: A pilot study. *J. Clin. Med.* **12**10.3390/jcm12041586 (2023). 10.3390/jcm12041586 PMC9962489 36836121

[91] Kammerlander, A. A. et al. Feature tracking of global longitudinal strain by using cardiovascular MRI improves risk stratification in heart failure with preserved ejection fraction. *Radiology* **296**, 290–298. 10.1148/radiol.2020200195 (2020). 32484413 10.1148/radiol.2020200195

[92] Lim, C. et al. Quantification of myocardial strain assessed by cardiovascular magnetic resonance feature tracking in healthy subjects-influence of segmentation and analysis software. *Eur. Radiol.* **31**, 3962–3972. 10.1007/s00330-020-07539-5 (2021). 33277669 10.1007/s00330-020-07539-5 PMC8128822

[93] Nazir, S. A. et al. Inter-study repeatability of circumferential strain and diastolic strain rate by CMR tagging, feature tracking and tissue tracking in ST-segment elevation myocardial infarction. *Int. J. Cardiovasc. Imaging*. **36**, 1133–1146. 10.1007/s10554-020-01806-8 (2020). 32152811 10.1007/s10554-020-01806-8 PMC7228913

[94] Hiramatsu, T. et al. Liraglutide relieves cardiac dilated function than DPP-4 inhibitors. *Eur. J. Clin. Invest.* **48**, e13007. 10.1111/eci.13007 (2018). 30054920 10.1111/eci.13007 PMC6175244

[95] Ko, K-Y. et al. Longitudinal evaluation of myocardial glucose metabolism and contractile function in obese type 2 diabetic Db/db mice using small-animal dynamic 18 F-FDG PET and echocardiography. *Oncotarget* **8** (2017). 10.18632/oncotarget.21202 PMC5675673 29152121

[96] Shao, X. et al. Dynamic evolution and mechanism of myocardial glucose metabolism in different functional phenotypes of diabetic cardiomyopathy - a study based on (18) F-FDG micropet myocardial metabolic imaging. *Diabetol. Metab. Syndr.* **15**, 64. 10.1186/s13098-023-01038-5 (2023). 37005683 10.1186/s13098-023-01038-5 PMC10067248

[97] Hellman, R. Glycemic variability in the use of Point-of-Care glucose meters. *Diabetes Spectr.* **25**, 135–140. 10.2337/diaspect.25.3.135 (2012).

[98] Nahra, R. et al. Effects of cotadutide on metabolic and hepatic parameters in adults with overweight or obesity and type 2 diabetes: A 54-Week randomized phase 2b study. *Diabetes Care*. **44**, 1433–1442. 10.2337/dc20-2151 (2021). 34016612 10.2337/dc20-2151 PMC8247525

[99] le Roux, C. W. et al. Glucagon and GLP-1 receptor dual agonist survodutide for obesity: a randomised, double-blind, placebo-controlled, dose-finding phase 2 trial. *Lancet Diabetes Endocrinol.* **12**, 162–173. 10.1016/S2213-8587(23)00356-X (2024). 38330987 10.1016/S2213-8587(23)00356-X

[100] Nalisa, D. L. et al. Efficacy and safety of mazdutide on weight loss among diabetic and non-diabetic patients: a systematic review and meta-analysis of randomized controlled trials. *Front. Endocrinol. (Lausanne)*. **15**, 1309118. 10.3389/fendo.2024.1309118 (2024). 38440786 10.3389/fendo.2024.1309118 PMC10911117

[101] Altimmune announces positive results from week 24 interim analysis of Pemvidutide MOMENTUM phase 2 obesity trial and 12-Week phase 1b type 2 diabetes safety trial. (2024).

[102] Jastreboff, A. M. et al. Triple-Hormone-Receptor agonist Retatrutide for Obesity - A phase 2 trial. *N Engl. J. Med.* **389**, 514–526. 10.1056/NEJMoa2301972 (2023). 37366315 10.1056/NEJMoa2301972

[103] Gejl, M. et al. Exenatide alters myocardial glucose transport and uptake depending on insulin resistance and increases myocardial blood flow in patients with type 2 diabetes. *J. Clin. Endocrinol. Metab.* **97**, E1165–1169. 10.1210/jc.2011-3456 (2012). 22544917 10.1210/jc.2011-3456

[104] Gejl, M. et al. Influence of GLP-1 on myocardial glucose metabolism in healthy men during Normo- or hypoglycemia. *PLoS ONE*. **9**, e83758. 10.1371/journal.pone.0083758 (2014). 24400077 10.1371/journal.pone.0083758 PMC3882300

[105] Lepore, J. J. et al. Effects of the Novel Long-Acting GLP-1 Agonist, Albiglutide, on Cardiac Function, Cardiac Metabolism, and Exercise Capacity in Patients With Chronic Heart Failure and Reduced Ejection Fraction. *JACC Heart Fail.* **4**, 559–566. 10.1016/j.jchf.2016.01.008106 (2016). 10.1016/j.jchf.2016.01.008 27039125

[106] Nielsen, R. et al. Effect of liraglutide on myocardial glucose uptake and blood flow in stable chronic heart failure patients: A double-blind, randomized, placebo-controlled LIVE sub-study. *J. Nucl. Cardiol : official publication of the Am. Soc. Nucl. Cardiol.*10.1007/s12350-017-1000-2107 (2017). 10.1007/s12350-017-1000-2 28770459

[107] Chen, W. J. Y. et al. Effects of exenatide on cardiac function, perfusion, and energetics in type 2 diabetic patients with cardiomyopathy: a randomized controlled trial against insulin glargine. *Cardiovasc. Diabetol.* **16**, 67. 10.1186/s12933-017-0549-z (2017). 10.1186/s12933-017-0549-z PMC5438489 28526033

[108] Mather, K. J. et al. Combination GLP-1 and Insulin Treatment Fails to Alter Myocardial Fuel Selection vs. Insulin Alone in Type 2 Diabetes. *J. Clin. Endocrinol. Metab.* **103**, 3456–3465. 10.1210/jc.2018-00712 (2018). 10.1210/jc.2018-00712 PMC6126889 30020461

